# Novel hybrid treatment for extensive aortic arch aneurysms, a case report

**DOI:** 10.1186/s13019-021-01453-7

**Published:** 2021-04-13

**Authors:** Mixia Li, Hulin Piao, Yong Wang, Kexiang Liu

**Affiliations:** Department of Cardiovascular Surgery, The Second Hospital of Jilin University, N0.218 Ziqiang Street, District Nanguan, City Changchun, Province Jilin China

**Keywords:** Extensive aortic arch aneurysm, Single-stage surgery, Hybrid approach

## Abstract

**Background:**

The treatment for extensive aortic arch aneurysms involving the aortic arch and descending aorta is challenging for most cardiovascular surgeons. The surgical treatment is associated with a very high mortality rate. The optimal treatment has not been defined.

**Case presentation:**

A 49-year-old male was hospitalized due to chest and upper back pain. Computed tomography angiography (CTA) demonstrated there was an extensive aortic arch aneurysm extending to the left common carotid artery and descending aorta. A novel single- stage hybrid surgery was performed on the patient through two steps: treatment of the aortic arch through median sternotomy and thoracic endovascular artery repair. The patient recovered uneventfully.

**Conclusions:**

Our single-stage hybrid repair approach is safe, simple and effective. It provides an alternative treatment for extensive aortic arch aneurysms.

## Background

Extensive aortic arch aneurysms involving the aortic arch and descending aorta is challenging for surgeons and the 5-year survival rate for patients without surgical treatment is only 13% [1, 2]. The optimal management of extensive aortic arch aneurysm involving the aortic arch and descending aorta has not been well established. The therapeutic options for this disease mainly includes single-stage open surgery, two-stage total arch replacement (TAR) with thoracic endovascular artery repair (TEVAR), and hybrid endovascular repair surgery.

## Case presentation

**Fig. 1:**
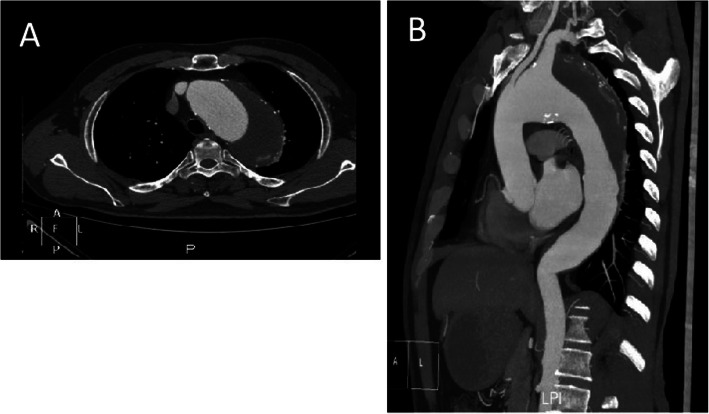
**a**, **b**: Preoperative CTA

A 49-year-old male with a history of severe hypertension was admitted due to chest and upper back pain. His CTA showed an extensive aortic arch aneurysm involving the left common carotid artery (LCCA) and descending aorta. The aneurysm’s maximum diameter was 67.8 mm, and the left subclavian artery was 27 mm in diameter (Fig. 1a,b). Given that the aneurysm involved the aortic arch, LCCA and descending aorta, a novel single-stage hybrid surgery was performed. The surgery consisted of two steps: treatment of the aortic arch through median sternotomy and thoracic endovascular artery repair (TEVAR).

**Fig. 2:**
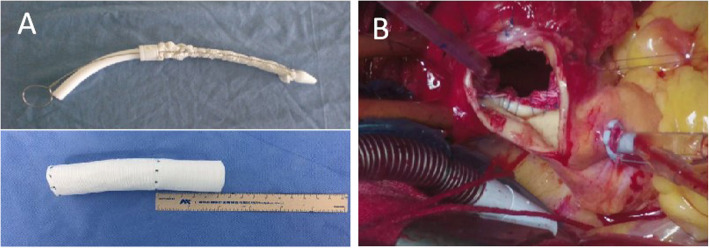
**a**: The stent-graft consisted of a 10 cm stented graft and a 5 cm proximal Dacron vascular prosthesis; **b**: Tightly suture proximal part of artificial vascular graft to aorta wall

**Fig. 3:**
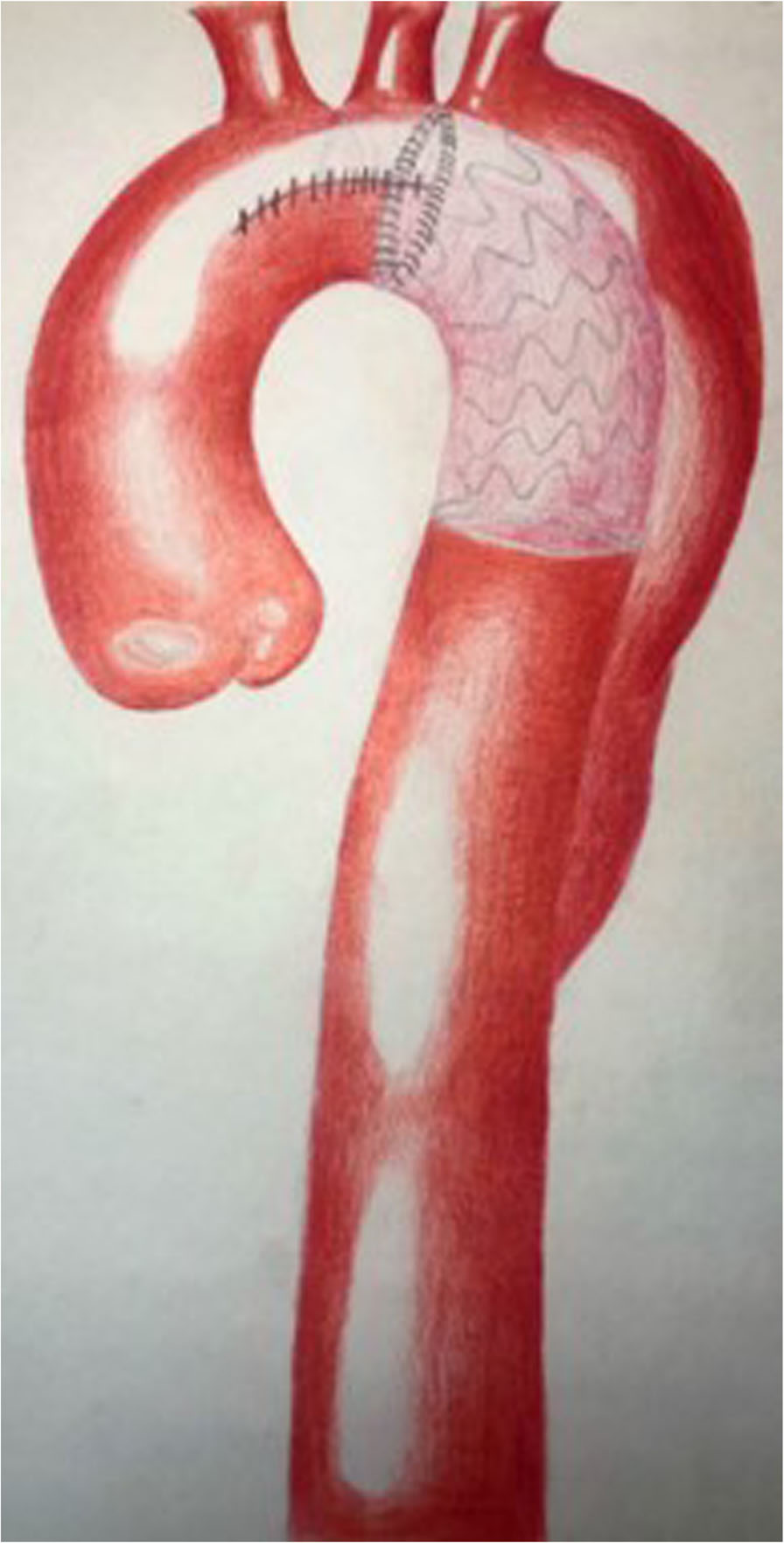
The treatment of the aortic arch was completed

After general anesthesia, the patient’s back surface cooling began. A median sternotomy was performed. Cardiopulmonary bypass (CPB) was instituted through cannulations of the right femoral artery, the innominate artery, and the superior and inferior vena cava. Circulatory arrest was initiated when the rectal temperature reached 28 °C. After a transverse incision of the aortic arch wall between the orifice of imonimate artery and distal to left common carotid artery orifice, antegrade selective cerebral perfusion was started via innominate artery and LCCA cannulations. The proximal end of left subclavian artery was ligated. After the intraoperative assesment, a 30 mm stent-graft (Microport Medical Co. Ltd., Shanghai, China) (Fig. 2a) was inserted into the descending aorta. The proximal stent-free vascular graft was then retracted back into the aortic arch. Running 4–0 Prolene sutures were made between the prosthesis and normal tissue of the aortic arch distal to the LCCA ostium (Fig. 2b), which attached the vascular graft with the aortic arch. The incision of the aortic arch was then closed with 4–0 Prolene sutures. This completed the aortic arch repair (Fig. 3). Then CPB was gradually resumed and rewarming was started. An 8 mm prosthetic vascular graft was placed from the ascending aorta to the left axillary artery to reconstruct the left subclavian artery. Afterwards, a guidewire and a 6-French short sheath were introduced through the right femoral artery. The covered stent-graft (Valiant Captivia VAM3232 C200TE Medtronic, Inc., Minneapolis, Minneasota, USA) was advanced into the previously inserted stent-graft, which fully excluded the descending aneurysm. The rest of surgery was completed routinely. The durations for circulatory arres, cross-clamping, CPB time and TEVAR were 24, 36, 110, and 22 mins, respectively.

**Fig. 4:**
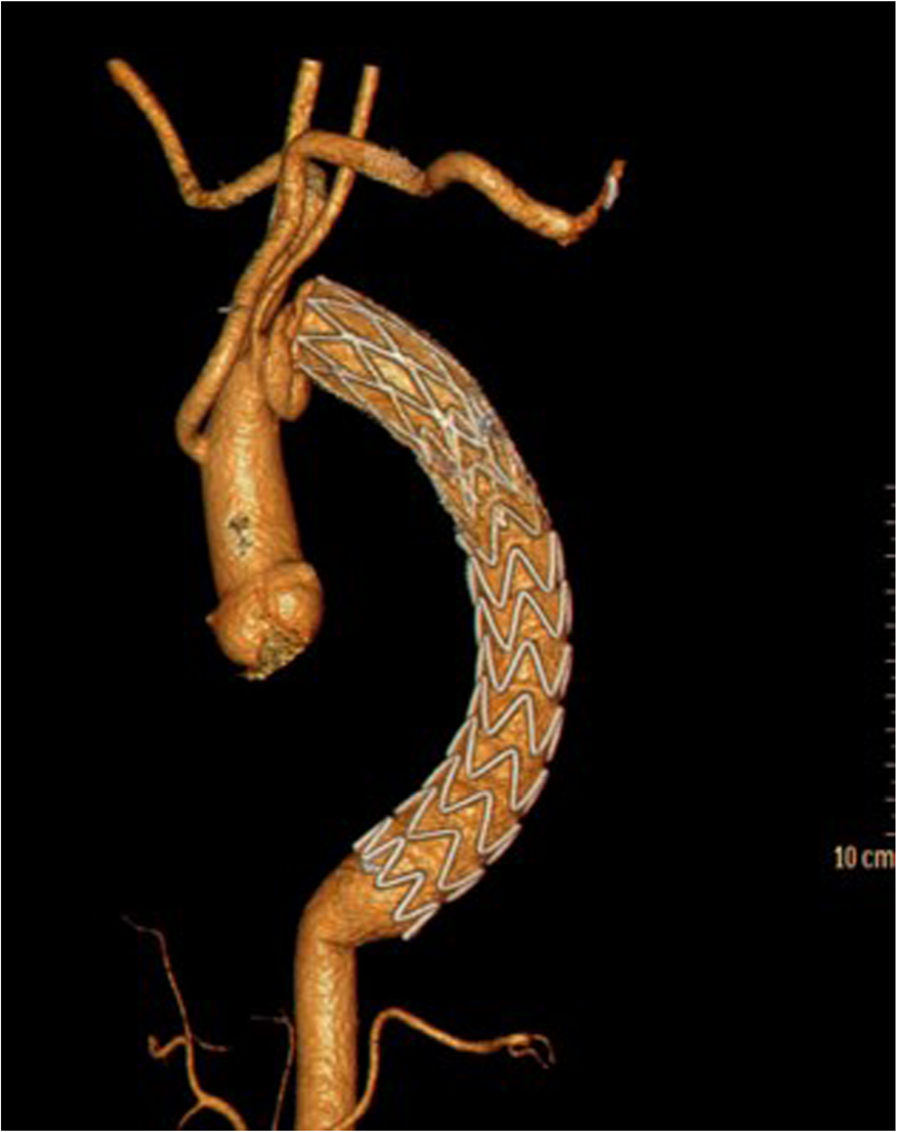
Postoperative CTA

The postoperative course was uneventful without neurologic deficits. CTA showed there were no endoleaks and no stent-graft displacement on day 14 postoperatively (Fig. 4). No blood transfusion was required during hospitalization.

## Discussion

The optimal surgical management for extensive aortic arch aneurysms involving the aortic arch and descending aorta has not been clearly established. Currently, proposed management includes single-stage open surgery [3], two-stage TAR with TEVAR, and hybrid endovascular repair [1]. Single-stage open surgery requires the exposure of the entire thoracic aorta, such as in a median sternotomy with left thoracotomy. The extensive surgery is invasive, and requires longer CPB time and longer cerebral protection time. These factors increase the risk of serious complications. Patients with multiple comorbidities may experience significant morbidity and mortality from both neurologic and cardiovascular complications [4].

The two-stage TAR with TEVAR can avoid extensive exposure, but it requires the replacement of the aortic arch and the anastomosis of supra-aortic vessels. The distal anastomosis on the aortic arch is deep and must be conducted on diseased aortic wall. This is a technical challenge and increases the risk of unmanageable bleeding. The replacement of the aortic arch also increases the number of anastomoses. The branched graft may be twisted and occluded [5].

Hybrid endovascular repair, including supra-aortic vessel transposition combined with TEVAR, has emerged as an alternative, to reduce the complications of open surgical repair. However, it is still associated with multiple complications. It has been reported that the incidences of stroke and endoleak were 9.7–14.3% and 19–24.5%, respectively [6, 7]. The long-term outcome of this hybrid treatment has not been reported.

Compared with the above techniques, our approach has avoided the aortic arch distal anastomosis. We repaired the aortic arch from inside the aortic arch, which simplifies the surgical procedures and avoids unmanageable bleeding. Unlike hybrid endovascular repair, our approach has securely sutured the stent-graft with normal tissue of the aortic arch. As a result, our approach avoids endoleak and stent-graft displacement. Moreover, the stent-graft can provides enough landing zone for TEVAR, ensuring coverage of the entire aneurysm. The patient fully recovered without any complications.

## Conclusions

Our single-stage hybrid repair approach is safe, simple and effective. It is an alternative treatment for extensive aortic arch aneurysms involving the aortic arch and descending aorta.

## Data Availability

Data sharing not applicable to this article as no datasets were generated or analyzed during the current study.

## Abbreviations

CTA: Computed tomography angiography
TAR: Total arch replacement
TEVAR: Thoracic endovascular artery repair
LCCA: Left common carotid artery
LSA: Left subclavian artery
RCCA: Right common carotid artery
IA: Innominate artery
SG: Stent graft
CPB: Cardiopulmonary bypass
